# Switzerland’s Narcotics Regulation Jungle: Off-Label Use, Counterfoil Prescriptions, and Opioid Agonist Therapy in the French-Speaking Cantons

**DOI:** 10.3390/ijerph182413164

**Published:** 2021-12-14

**Authors:** Caroline Schmitt-Koopmann, Carole-Anne Baud, Valérie Junod, Olivier Simon

**Affiliations:** 1 Service of Addiction Medicine, Lausanne University Hospital (CHUV) and University of Lausanne, CH 1004 Lausanne, Switzerland; olivier.simon@chuv.ch; 2 Faculty of Business and Economics, University of Lausanne, CH 1011 Lausanne, Switzerland; caroleanne.baud@unil.ch (C.-A.B.); valerie.junod@unil.ch (V.J.); 3 Faculty of Law, University of Geneva, CH 1211 Genève, Switzerland

**Keywords:** public health, administration, regulation, narcotics, opioids, controlled medicines, opioid agonist treatments, off-label, counterfoil prescriptions, opioid informed consent

## Abstract

The word “narcotic” is often first associated with “illicit drugs”. Yet, many “narcotic” and psychotropic substances are, in fact, medicines. Controlled medicines (CM) are products that meet the legal definition of both a “narcotic” under the Swiss Narcotics Act and of a medicine under the Therapeutic Products Act. We aim to examine how similar and how different, respectively, the implementation of CM regulations is throughout French-speaking Switzerland. Based on a legal analysis of the cantonal regulations, we conducted semi-structured interviews with cantonal pharmacists and cantonal physicians. We asked them how they perceive and implement the federal legal requirements. We find that some of these requirements have fallen into disuse, notably the federal duty to notify off-label use of CM. We observe that counterfoil prescriptions in their current paper format are a veritable data graveyard in the sense that they are not actively used to monitor or supervise the market. Moreover, we detect different conditions for opioid agonist treatment authorization. Some cantons require additional physicians’ training or written commitments by the person treated. Our mapping of the CM regulation implementation can serve as a basis for cantons to review their practices.

## 1. Introduction

In Switzerland, controlled medicines (CM) are products that meet the legal definition of both a “narcotic” under the Federal Narcotics Act (Betäubungsmittelgesetz, BetmG) [1] and of a medicine under the Federal Therapeutic Products Act (Heilmittelgesetz, HMG) [2]. Hence, CM must abide by the rules in both statutes. Moreover, the implementation of CM regulation is made more complex due to the federalist nature of Switzerland (divided into 26 cantons) [3]. In particular, cantons have the obligation to implement the federal regulation, whereas their competence to impose additional requirements is subject to debate.

CM are widely used substances [4,5] that, like all medicines, are rigorously tested before being approved by the Agency for Therapeutic Products (Swissmedic) in Switzerland. They include many substance classes and cover a large variety of therapeutic indications and can be used in pain management, anesthesia, palliative care, and psychiatry [6]. For example, morphine, the quintessential opioid analgesic, relieves moderate and severe pain [7], while long-acting opioid agonists, such as methadone, play a central role in treating opioid dependence syndrome. Another class of widely used CM is benzodiazepines, which have anxiolytic, anticonvulsant, sedative and sleep-inducing properties [8]. According to the International Narcotics Control Board estimation, in 2021, the 8.6 million Swiss inhabitants will use 3277 kg morphine, 7176 kg methadone, 5000 kg clonazepam and 5500 kg methylphenidate [9,10,11].

In Switzerland, all controlled substances, including CM, are classified into different schedules [6]. Schedule A includes CM, such as opioids and amphetamines, while benzodiazepines, “z-drugs”, and barbiturates are schedule B substances. Schedule D contains prohibited substances, such as tetrahydrocannabinol and diamorphine. The other schedules are preparations with low doses (C), raw materials (E), precursors (F), and auxiliary chemicals (G).

The BetmG is meant to strike a reasonable balance between preventing substance trafficking and ensuring access to care. It is often revised, with the last major change dating to 2011 when the four-pillar policy (prevention, therapy, harm reduction, repression) and treatment with diamorphine prescription (pharmaceutical heroin) were enshrined in the law. In May 2021, a new article in the BetmG went into force that allows “recreational” use of cannabis in canton-led pilot tests. Two ordinances of the BetmG further detail how the BetmG is to be implemented. The Narcotics Control Ordinance (BetmKV) [12] specifies the controls exercised over the CM market. In contrast, the Narcotics Addiction Ordinance (BetmSV) [13] addresses prevention, harm reduction, and treatment of individuals suffering from opioid dependence syndromes.

The BetmG and its ordinances charge the cantons with various duties and implementing obligations [14]. Among them, and carried out by the cantonal physicians (CPY) or cantonal pharmacists (CPA), are the duties to:

1. Receive notifications of CM prescriptions for indications other than those for which they were authorized (so-called off-label use; Art. 11 para. 1bis BetmG);
2. Provide counterfoil prescription blocks to physicians intending to prescribe specific CM (Art. 47, para 5 BetmKV);
3. Grant prior authorization for prescribing, dispensing, and administering CM to treat persons “dependent on narcotics” (i.e., authorization of opioid agonist treatments (OAT); Art. 3e BetmG).

This study aims to analyze the cantonal implementation of these three aspects of federal CM legislation for which cantons are responsible. We investigate these areas since information on how these tasks are implemented is not available in public sources. A specialty of the Swiss system is that practices can differ between cantons due to the federalist structure of Switzerland. Furthermore, CPY/CPA have not only executive power but can shape practices. The only way to gather information on how the BetmG is implemented is by asking CPY and CPA.

The three above mentioned obligations are detailed below.

1. A medicine is used off-label in Switzerland when the physician prescribes it in a manner not foreseen by the professional product information approved by Swissmedic. Off-label use is not prohibited as such and is common and routine in many medical sectors (e.g., oncology and pediatric medicine) [15,16,17]. Physicians must be sure to obtain the treated person’s prior free and informed consent. In other words, individuals treated must know that they are receiving an off-label treatment and that the latter may carry certain increased risks [18,19]. Generally, off-label use of medicines does not require any notifications.
  However, this is different for CM, for which prescription for non-approved indications must be reported to the relevant cantonal authorities within 30 days (Art. 11 para. 1bis BetmG). As this applies to all CM (e.g., benzodiazepines), we expect the cantons to receive many off-label notifications.
  The number of notifications would depend, in part, on whether a narrow or broad interpretation of off-label use is retained. Narrow off-label use relates only to the therapeutic indication (i.e., a medicine used for a medical condition that has not been approved). Broad off-label use includes all deviation from the approved notice of use (professional information), such as use at a greater or lower dosage, for a longer or shorter period than authorized, or in other patient populations [18,20].
  It is not clear whether Art. 11 para. 1bis BetmG requires notification only for narrow off-label use or also for broad off-label use. During a consultation of the initial article, which spelled out a broad definition, a majority of cantons were critical of the initial version [21,22,23]. They drew attention to the additional work for physicians, questioned the benefit of the article, and stated the unclear consequences for cantonal and federal administrations [22]. Yet, the current ordinance would support a broad interpretation since notification must include the name of the CM, quantity, strength, and indication [24]. Hence, we expect cantons to use different definitions.
2. One measure specified in the BetmKV to control the CM market is the requirement imposed on physicians to prescribe Schedule A and D substances only on triplicate counterfoil prescriptions [25]. In contrast, regular prescriptions are sufficient for Schedule B CM [26]. Swissmedic supplies the cantons with the trilingual counterfoil prescription block (CPB; French, German, and Italian) [27]. Each CPB consists of 25 counterfoil prescriptions featuring a copy protection safety mark and a unique prescription number. Cantons give or sell CPB to physicians. Hence, cantons may track stolen or lost CPB. Each counterfoil prescription consists of one original and two copies (counterfoils). The original (white) is redeemed at the pharmacy, while the physician must keep the blue foil, and the red foil is destined for the insurance company [28]. This counterfoil system creates a paper trail of dispensed CM. We would expect similar cantonal administrative procedures regarding the provision and use of CPB.
3. Opioid agonist treatment (OAT) is the standard treatment for opioid dependence syndrome. Its efficacy has been proven in multiple studies [29,30,31]. Opioid agonists, such as methadone, create a state of protective tolerance, which allows the person in OAT to reduce or stop taking heroin and initiate other medical and psychosocial care in parallel [32]. According to Art. 8 BetmSV, OAT aims to create distance from the “drug scene,” prevent procurement crime, decrease high-risk forms of consumption, reduce substance use and initiate abstinence.
  In Switzerland, all OAT treatments must be authorized by the cantons [33]. The BetmSV further specifies that only qualified persons can provide OAT [34], but only physicians can ask for authorization [35]. Apart from their profession, the type of qualification is not further specified in the federal legislation and is absent in most cantonal regulations. We would expect that all physicians with a license to practice (according to Art. 9 BetmG) are considered to have the qualifications necessary to provide OAT. Based on the supposedly exhaustive nature of federal legislation, we hypothesize that there are only minor variations in the conditions for OAT authorization.
  We selected physicians’ training and written commitments to comply with specific modalities by a person in OAT (so-called “treatment contracts”) since an all-encompassing analysis of OAT authorizations, in general, would have extended beyond the limits of this article.

There have been very few studies regarding this subject, and research so far has only addressed the implementation of select provisions of CM regulation. For instance, a report investigated exceptional licenses of prohibited controlled substances (schedule D) for restricted medical use [36]. Yet, this is a federal task and is therefore out of scope for this study. A 2013 study of OAT regulation, including Switzerland, highlighted differences between some cantons [37]. The study found that cantonal provisions, whether recommendations or legally binding, are often particularly detailed concerning OAT. However, the laws and directives have been revised since, and only two of the cantons from our study were analyzed. Another more recent study reviewed OAT clinical practices and found that OAT practices vary significantly across and within countries [38]. Switzerland was not included in this review. Still, it is reasonable to assume that federalist Switzerland also has in-country variation. Finally, a report published by the Pompidou Group established guiding principles and emphasized the duty of countries to ensure a coherent framework for OAT [39]. However, a status quo description would be necessary to study whether Switzerland complies with these guiding principles.

Our research is, to our knowledge, the first attempt to describe the implementation of CM regulation overall at a cantonal level. It will allow cantons to compare their practices and evaluate them. The study is part of a research project funded by the Swiss National Science Foundation (SNSF grant number 182477), which aims to analyze the current CM legislation in Switzerland.

## 2. Materials and Methods

We interviewed the CPY and CPA of French-speaking Switzerland for our study since they are responsible for implementing the three areas listed above. Additionally, CPY and CPA shape the implementation through their surveillance and advisory roles.

In preparation for the interviews, we identified the relevant cantonal legal texts, in force at the time of the analysis, using the internet platform www.lexfind.ch (last accessed on 27 September 2021) and the online available cantonal legal collections (keyword search: “Stupéfiant” and “Addiction/Dépendance”) (see supplementary materials, document S1: legal bases & documents, pp. 4–5). Furthermore, we analyzed guidelines and recommendations regarding OAT and CM, which were available on the websites of the CPY and CPA. We base our analysis on regulation in force at the time of evaluation (2020–2021). However, half of the cantons (Valais, Vaud, Geneva, Switzerland) report that they are in the process of rewriting their directives.

Based on the collected legal texts, we wrote a 10-page interview guide, which we adapted for each canton, considering the publicly available information for each canton (see supplementary materials, document S1: interview guide, pp. 1–2). The questions cover how the CPY/CPA choose to implement the legal requirements. After a warm-up on their experience in the role, and team size, we asked about the cantonal practices. We asked participants if they receive off-label use notifications, who analyzes the notifications, and how. For counterfoil prescription blocks, we asked participants if there are limits on the number of blocks, and who is responsible for supplying and tracking them. For OAT authorizations, we asked the CPY how they receive the requests and which additional conditions they impose (e.g., written commitment by the person in OAT).

The participants were interviewed one-on-one in semi-structured interviews ranging in duration from one to one and half-hours. We conducted the interviews with the CPY and CPA separately to avoid cross-influences [40]. This setup also guaranteed the anonymity of responses. If the participant explicitly consented, the interviews were audio-recorded to facilitate the creation of a transcript for data analysis.

After the interview, the transcript was completed and finalized. We continuously coded (structural codes) and analyzed the interview transcripts using the MaxQDA software [41] with the iterative five-step procedure described by Tolley et al. [42]: (1) reading the entire transcript; (2) coding using structural codes (aligned with specific questions from the interview guide); (3) displaying the data using coding reports and creating memos for each main code; (4) reducing the data; (5) interpreting the data.

For the analysis, we decided on an analytical framework approach with qualitative content analysis [43,44,45,46]. We ran a deductive manifest analysis using a categorization matrix since our research aims to describe differences and similarities between cantons. To allow the creation of codes we had not anticipated based on our legal analysis, we selected an unrestrained categorization matrix [46] (see supplementary materials, document S1: data & analysis, p. 3).

The research ethics committee of the University of Lausanne (Commission d’éthique de la recherche) approved the research protocol. Each participant was informed about the aim of the study and gave written consent to participate.

## 3. Results

We base our results on twelve interviews with each CPA and CPY or their deputies of the cantons Geneva (GE), Fribourg (FR), Jura (JU), Neuchatel (NE), Valais (VS), and Vaud (VD).

### 3.1. Off-Label Notification

As explained above, physicians who prescribe a CM off-label are supposed (more precisely legally required) to notify the cantonal authority. However, all interviewed CPY and CPA reported that they rarely receive notifications; neither do they enforce the provision even though Art. 21 BetmG clearly states that any person who willfully fails to file reports under Articles 11 para. 1bis is liable to a custodial sentence not exceeding three years or a monetary penalty. Moreover, an offender is liable to a fine if he or she acts through negligence.

Asked, CPY/CPA from all cantons said they defer to a broad definition of off-label use. Some cantons observed that receiving notifications would lead to unmanageable paperwork and an ultimately excessive workload. Three CPA/CPY expressed that they felt the provision to be useless in the sense that it had no clear assigned objective and that notifications could not be put to any use, given the lack of access to the medical file.

Neuchatel is the only canton in our study with a form on their website to handle such notifications [47]. Nevertheless, Neuchatel generally only receives notifications for significant deviations. Furthermore, they do not receive MUC off-label notifications for palliative care, where off-label use is common. Currently, Neuchatel receives most notifications from pharmacists for persons in OAT. Physicians seem to be reluctant to comply with the federal provision. Neuchatel plans to develop its processes regarding CM off-label use notifications further. The CPA will ask a person with high doses of off-label CM to sign a therapeutic commitment document, which restricts the prescription and dispensation of medicines to a single physician and pharmacy [48].

Two CPA/CPY expressed interest in receiving off-label notifications, while one CPA/CPY responded not wanting them. Cantons admitted that they had no established processes in place to react to notifications other than archiving them. However, some cantons reported that they might require explanations from prescribers or a second medical opinion when notified of extremely high doses of CM. Overall, CPY and CPA appear comfortable with the physicians’ non-compliance with this federal provision.

### 3.2. Counterfoil Prescription Blocks

The responsibility to provide CPB to physicians is allocated differently: in Vaud, Geneva, and Jura, CPY sells the CPB, while in the other cantons, it is the CPA. All cantons have some form of tracking system in place for CPB. Vaud reports to send out around 2550 CPB per year, assuming it takes about 5 min to enter data into their tracking system and put the CPB in the post, it would take one full-time employee around five weeks just filling CPB requests.

The prices vary widely, from being free in the canton of Vaud to just over CHF 30 in Geneva (see Table 1). It is unclear how these prices are determined, but Geneva splits their price into a price per block and administrative fees. Physicians cannot invoice the treated person or insurers for the cost of the CPB; they ultimately bear this expense.

There is no large-scale follow-up of the counterfoil prescriptions once the CM has been dispensed at the pharmacy. However, occasionally some cantons collect data and conduct statistical investigations for certain substances using counterfoil prescriptions [49,50]; most study results remain unpublished.

### 3.3. Opioid Agonist Treatments: Physician Training and “Treatment Contracts”

All six cantons have issued detailed directives on OAT, which differ in some aspects. The oldest directive (VD) dates back to 2010 and the newest to 2018 (FR). Conditions for OAT authorization differ substantially in Fribourg and Valais compared to the other cantons.

**Figure 1:**
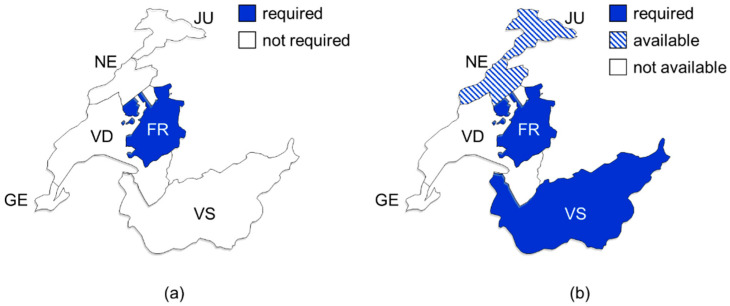
Maps showing requirements for opioid agonist treatments regarding specific physicians’ training (**a**) and written commitment by a treatment-seeking person to comply with specific modalities (**b**). FR, Fribourg; GE, Geneva; JU, Jura; NE, Neuchatel; VS, Valais; VD, Vaud.

Indeed, Fribourg is currently the only canton, which requires OAT prescribing physicians to undergo an initial half-day course organized by the CPY (see Figure 1a). Physicians can only prescribe OAT after attending the training and must attend further training once every two years. The effectiveness of these courses is not tracked. The other cantons expressed interest in proposing, but not requiring, a specific online training on OAT for physicians.

Valais is the only canton that requires the inclusion of a third entity for OAT, the addiction counselor. Every person in OAT in Valais must accept the socio-medical approach of the Addiction Valais Foundation, which employs the counselors. The assigned addiction counselor will accompany a person in OAT from the beginning.

The cantonal authorities of both Valais and Fribourg require so-called “treatment contracts” for OAT authorization (see Figure 1b) [51,52]. The physicians can choose to impose the CPY-issued document in Jura and Neuchatel. In Vaud and Geneva, the CPY do not promote these written commitments. These documents are a mix of rules of conduct for the person in OAT, combined with patient information; they contain vague statements of consequences in case of non-adherence by the person in OAT. While these documents describe in detail the responsibilities of the person in OAT, the responsibilities of the treating physician and dispensing pharmacist are only described in one written commitment (Jura).

All four documents contain passages stating that the use of alcohol or other substances together with OAT can have undesirable effects; that the treated person must inform the treating physician of any other medicines he is taking; that the person in OAT will announce absences from OAT dispensation early; and that lost, stolen, or vomited doses are not replaced [53,54,55,56]. Furthermore, all four documents impose that the person in OAT has to accept urine tests when demanded by the physician. Signatories of these written commitments are the person in OAT, the treating physician, and the pharmacist. In Valais, additionally, the addiction counselor must sign the so-called “multipartite contract.”

## 4. Discussion

The three areas described above have their origin in federal legislation. Therefore, we would expect the cantonal interpretation and implementation to be similar in all six cantons. Yet, our study reveals that this is not the case. All six cantons are similar in that they do not receive nor enforce off-label notifications, but they differ in CPB pricing. Additionally, two cantons deviate in their conditions for OAT authorizations. They require written commitments to be submitted for authorization (Fribourg and Valais) and OAT training for physicians (Fribourg).

The lack of enforcement of off-label notifications and the corresponding disregard for a binding federal provision is unexpected. The logical explanation for cantons not enforcing the notification is that the cantons do not see any benefit in doing so. Moreover, the volume of notifications would likely be extremely high. It is estimated that 20–60% of all treatments are off-label [57]. CPA/CPY reported that they would be unable to manage off-label notifications with their current resources. Despite sanctions for non-compliance being custodial sentences or monetary penalties [58], to our knowledge, no penalty has been imposed.

As for the definition, it is puzzling that despite being in force for ten years, there is still no consensus on what constitutes CM off-label use as per Art. 11 para. 1bis BetmG. Widely accepted is the broad definition of off-label use issued by the cantonal pharmacist’s association [18]. However, their document does not elaborate on CM off-label use.

Based on the review of the six cantons (pending the investigation of the other cantons) maintaining the provision should be questioned.

CPB, whilst implemented slightly differently, pose an administrative burden for the responsible service and canton overall. We hypothesize that the cantons charge for CPB to cover the administrative costs this way. Nevertheless, the reasons behind the pricing differences and their effects on the health system need further investigation. Physicians could be dissuaded from prescribing certain CM if they perceive the process of ordering CPB to be unduly cumbersome or expensive. Moreover, it is unfortunate that the data resulting from counterfoil prescriptions is barely, if at all, exploited. We believe this is likely due to the paper format. It discourages the detection of trends and prescription patterns. Thus, in their current format, counterfoil prescriptions are veritable data graveyards. Hence, alternatives that alleviate the administrative burden for the cantons and enable easy monitoring should be evaluated (e.g., integration into the electronic patient record [59]).

Several CPY are contemplating additional OAT training for physicians; this suggests that CPY believe that not all physicians are sufficiently qualified through their ordinary medical curriculum. This matches earlier findings, which identified a lack of training and information on OAT [60].

The use of “treatment contracts” is legally questionable, as persons in OAT do not have the choice to refuse them. Indeed, if they do refuse, they can be denied treatment. Moreover, neither federal nor cantonal legislation provides a basis for these documents. Thus, it remains unclear if the cantons have the competency to require such written commitments, as they might pose a constraint on access to treatment. Patient information, consent, and treatment plan are part of any medical treatment and are rarely written. Furthermore, the clauses forcing a person to accept urine tests after initiation of the treatment should be reviewed [61]. There is no scientific evidence that shows a positive effect of urine tests on OAT outcomes. Similarly, written commitments have only weak scientific evidence for effectiveness [62,63].

More generally, we believe that the diverging interpretation and implementation of CM regulation are historically grown and further explained by an irregular exchange between cantons. Despite the variation in implementation, our chosen methodology does not allow discerning outcome differences. However, research is needed on how the cantons can achieve the best outcomes.

While the BetmG is an old statute, it has undergone several revisions. Hence, it is striking that there are no mechanisms to monitor cantonal implementation and evaluate the results. Possible explanations are the ambiguity and unclear objectives of the BetmG, the existence of too many stakeholders and overlapping authorities, a lack of motivation, or even resistance from implementers.

CPA and CPY must accomplish a broad array of tasks that goes beyond CM regulation (e.g., SARS-COV-2 pandemic, physicians practice licenses, pharmacy inspections) with limited staff and financial resources. Yet, despite requiring considerable administrative efforts, CM regulation does not seem to be a priority for review for the authorities. We believe that it would be worthwhile to determine which tasks can be centralized or even eliminated. The freed resources could then be attributed, for example, to training.

This study has limitations that need consideration. Our study analyzes the practices of CPA and CPY but does not look at the effects of these practices in the field. Furthermore, our findings cannot be generalized since the six investigated cantons are not representative of Switzerland. With our study, we identified several areas that need further research: OAT authorization tools, urine tests in OAT, written commitments from people in OAT, alternatives to paper CPB, and the usefulness of CM off-label use notifications.

## 5. Conclusions

In conclusion, our research documents the implementation of Swiss CM regulation on a cantonal level for the first time. Our results show that the variation between cantons in implementation is more extensive than we would expect. We unveiled a need for a critical review of the practices resulting from CM regulation from both a legal and public health standpoint. By mapping the differing practices, our research could serve as a basis for cantons to review their implementation and compare themselves to other cantons.

## Figures and Tables

**Table 1:** Cantonal management of counterfoil prescription blocks (CPB) distribution. CPY and CPA stand for cantonal physician, respectively, and cantonal pharmacist.

Canton	Providing CPB	Price per CPB
Fribourg	CPA	CHF 7.80 **
Geneva	CPY	CHF 30.30 *
Jura	CPY	free
Neuchatel	CPY	free
Valais	CPA	CHF 10.00 **
Vaud	CPA	free

* One CPB costs CHF 25 plus CHF 5.30 for registered mail. ** Price includes mailing costs.

## Data Availability

The data presented in this study are available on request from the corresponding author. The data are not publicly available to maintain the anonymity of the participants. It is necessary to keep data confidential, even in anonymized format, since it might be comparatively easy to ascertain the participants’ identity. A re-identification waiver will be required to access the transcripts.

## Supplementary Materials

The following are available online at https://www.mdpi.com/article/10.3390/ijerph182413164/s1, Document S1: Supplementary information on the interview guide and methodology.
