# Supplementary material for: Switzerland’s Narcotics Regulation Jungle: Off-Label Use, Counterfoil Prescriptions, and Opioid Agonist Therapy in the French-Speaking Cantons
Source: Int J Environ Res Public Health. 2021 Dec 14;18(24):13164. doi: 10.3390/ijerph182413164 (PMC8700987; doi:10.3390/ijerph182413164)
Supplement: Supplementary file 1 [file ijerph-18-13164-s001.zip › ijerph-1417531-supplementary.pdf]

S1: Supplementary information on the interview guide and methodology

Interview guide

The interview guide we used covered areas we present in this publication (off-label notifications, counterfoil prescriptions, and OAT physicians training and "treatment contracts") and other areas. We only present the results from the pilot study, which was geographically limited to the French-speaking cantons of Switzerland. We will describe the interview guide and its creation further below.

We have intentionally not included the entire interview guide because the study has not yet been completed throughout Switzerland. The interview guide can be sent confidentially on request but should not be made publicly available in advance because it could influence the collective not yet interviewed (Swiss-German cantons and Ticino).

When we set out to determine how the BetmG is implemented and perceived by the Swiss Cantonal Physicians (CPY) and Cantonal Pharmacists (CPA), we chose a research design that started with a cantonal legal summary followed by interviews with CPA and CPY. We decided on semi-structured interviews, as that allowed the gathering of more details and nuances than a standardized questionnaire (paper or online) would have. Furthermore, we decided against a written format, as the participants would have likely self-censored themselves more. Alternatively, a multidisciplinary focus group format (bringing together people in treatment, professionals, and authorities). It was rejected because it is better suited to determine problems and perceptions of different stakeholders than to track the implementation of legislation.

We had two challenges to account for when designing the interview guide. The first is the limited amount of time available for the interview. CPA and CPY are included, to varying degrees, in the efforts to combat the SARS-COV2 pandemic and were on a tighter schedule than usual. The maximum time we could get to interview them was between 1h and 1h30min. This limited timeframe meant we would not be able to discuss all topics with each participant but rather discuss in detail the topics, which were their responsibility. The second challenge was that the cantons have allocated the tasks differently; this information was not always publicly available in a law paragraph or directive. Hence, in some cantons, the responsibility of tasks was only unveiled in the first interview. A good example is here also a result from our research: the responsibility for off-label notifications is allocated differently from one canton to the other. Furthermore, depending on the availability, we first interviewed the CPY and then CPA in some cantons or vice versa.
We have created a modular interview guide (Figure S1) to address these challenges. The modules cover all areas we wanted to discuss but allowed us to adapt quickly. Generally, we checked with each participant from each canton if they were responsible for the module, and if not, we moved on to another one. Nevertheless, this meant that the interview with the second participant was generally more straightforward because there was already less uncertainty regarding responsibility.

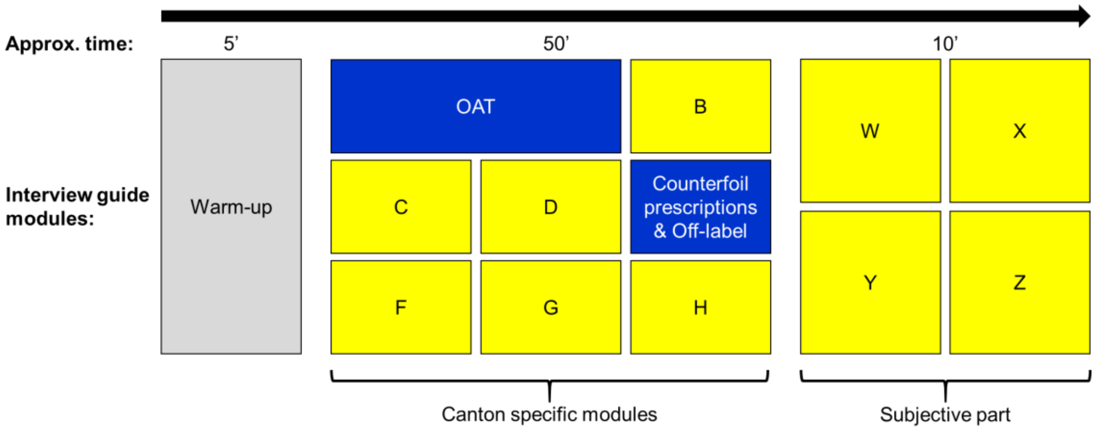

***Figure S1**: Graphic representation of the interview guide. The grey module represents questions that were asked to all participants. In contrast, depending on the cantons distribution of responsibilities, the participants were asked a specific combination of the blue and yellow boxes. The blue boxes are the modules from which we present results in this publication. The yellow boxes represent topics that are not covered in this publication.*

The evolution of the interview guide

We based the first interview guide on a legal analysis of the competencies given to the cantons. Our scientific advisory board, composed of former CPY/CPA and physicians, provided feedback, which we incorporated. For each task, we asked the participants; how do they interpret the law? How do they enforce and sanction it? What are the advantages and disadvantages?

The most significant change we made was to shift from asking about their interpretation of the legal articles to asking how they implement them in practice. Furthermore, we restructured the topics into modules and prioritized the most critical modules (e.g., OAT) by starting with those at the beginning. We also shifted all the questions regarding sanctioning into a separate topic instead of checking for each competence. We also quickly eliminated Art. 10 BetmKV because it was too broad a topic. We also removed the exact quantitative questions that participants might need to look up from the interview guide. To illustrate, we no longer asked them how many CPB they provide or sell in a year. Sometimes the participants volunteered this data. We will invite all CPY and CPA to a quantitative survey to gather comparative data across Switzerland.

The interview questions

There are more questions in the interview guide that we ask under each module. However, we do not share them here for the reasons mentioned above. The complete interview guide can be shared confidentially upon request.

Off-Label
According to Art. 11 para. 1bis BetmG: physicians and veterinary surgeons must report off-label CM use to the cantons.

- Who receives Off-label notifications?
- Do you receive such notifications?
    - How many do you receive?
    - From whom?
    - What do you do with them?
- If you don’t receive Off-label notifications: do you want them?
- What counts as off-label use?

Counterfoil-Prescriptions
Let us talk about counterfoil prescription blocks next.

- Who is responsible for providing/selling them to the physicians?
- How much do they cost?
- Is there a follow-up with counterfoil prescriptions?

OAT
I would now like to talk about substitution therapies. According to Article 3e BetmG and Articles 8 and 9 of the BetmSV, the cantons are responsible for issuing licenses for the dispensing, prescription, and administration of CM to treat dependence syndromes.

- Do prescribing physicians need to fulfill specific requirements to receive authorization for OAT?
    - Do they need to undergo specific training?
        - Who organizes the training? How long?
        - How often is it offered?
        - Is there follow-up training that is required?
        - Are pharmacists also invited?

In the cantonal document/directive/law “X," we found the mention of a "Therapeutic Contract."

- Do you require these for authorization?
    - If yes: Does it have to be submitted for authorization?
    - If no: Who decides whether a patient has to sign this “contract” then?
- Can persons in OAT opt-out?
- Can provisions be adapted?

Data & analysis

Transcripts

The interviews were conducted in French by CB and transcribed in French by VJ and CB. No translations were made because all authors are fluent.

Analysis of interviews

The interview transcripts were continuously coded and analysed using the following iterative procedure:

1. Reading the entire transcript;
2. Coding based on main codes (aligned with specific questions from the interview guide);
3. Displaying the data using coding reports and creating memos for each main code;
4. Reducing the data by creating matrix;
5. Interpreting the data.

For the analysis, we chose an analytical framework approach with content analysis. We decided on a mainly deductive approach because our research aims to describe differences and similarities between cantons. Furthermore, our analysis only describes what the participants actually said (manifest analysis). We used a preliminary matrix based on insights from the legal analysis that preceded interviews and structural codes to analyse the data. We chose an unrestrained categorization matrix to allow the creation of subcodes with inductive content analysis when necessary. In practice, the interviews were coded firstly with main codes, structural codes corresponding to questions in the interview guide, e.g., “off-label definition.” Then the text in the main codes was categorized, e.g., subcode “broad off-label definition.” If no suitable code existed, creating a new code with inductive content analysis was considered, e.g., subcode “off-label notifications by pharmacists instead of physician.”

Examples from coding and categorization

Here we show how the transcripts were coded and an excerpt of the categorization matrix (Figure S2). The transcripts and coding were in French, so the examples shown here are translations.

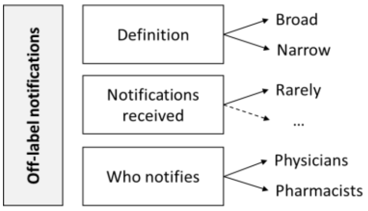

***Figure S2**: Illustration of the categorization matrix for the examples in the topic off-label with main codes (boxes) and subcodes (arrows).*

<u>Example 1: Topic Off-label, Code = **Off-label Definition**</u>
Corresponds to question: “What counts as off-label use?”

Participant #12:
“As soon as we leave the SwissmedicInfo context, it is off-label for us, whether it is the dose or the indication. We have taken this as a guideline even though we know that there are guidelines that indicate something other than the compendium.”
Subcode = **Broad off-label definition**

Participant #13:
“Indications and doses other than those provided for by Swissmedic must be notified.”
Subcode = **Broad off-label definition**

<u>Example 2: Topic Off-label, Code = **Number of Off-label notifications received**</u>
Corresponds to question: “Do you receive off-label use notifications?”

Participant #6:
“And yet I do not receive them. It's very rare that I receive them.”
Subcode = **Off-label notifications rare**

Participant #7:
“The law says that it should be announced spontaneously. This is never the case. This is never done. Zero. If a case is discovered, it is via pharmacies that notice a very high dosage. But not even then necessarily, we only see if the dosage is very, very high, but we don't see every case.”
Subcode = **Off-label notifications rare**
Subcode = **Off-label notifications by pharmacists instead of physicians**

Legal bases & documents used in the analysis

OAT directives

- FR - Richtlinien über die Substitutionsbehandlung bei Opiatabhängigkeit (betäubungsmittelgestützte Behandlung Suchtkranker) vom 21. August 2018
- GE - Directive sur la prise en charge medicamenteuse des personnes toxicodependantes du 1 mars 2012 (Entrée en vigueur : 1 juillet 2013)
- JU - Directives du médecin cantonal sur les traitements basés sur la substitution (TBS) avec méthadone, buprénorphine ou autres opioïdes en cas de dépendance aux opiacés du mai 2013
- NE - Recommandations du médecin cantonal concernant la prescription de stupéfiants destinés au traitement de personnes dépendantes 2017
- VD - Directives du Médecin cantonal concernant la prescription, la dispensation et l'administration des stupéfiants destinés à la prise en charge de personnes dépendantes (état le 01.05.2010)
- VS - Richtlinien des Departements für Gesundheit, Soziales und Kultur - Substitutuionsbehandlung von opioidabhängigen Personen vom 7. Januar 2016

Other CM-related cantonal documents

- FR - Behandlungsvertrag / Substitutionsbehandlung mit Betäubungsmitteln
- JU - Contrat thérapeutique multipartite fixant les modalités du suivi en officine des traitements par substitution aux opiacés
- NE - Contrat thérapeutique pour le traitement de substitution par des stupéfiants et/ou psychotropes off label use (benzodiazépines)
- NE - Off-Label Consentement Engagement
- NE - Recommandations concernant l'usage des benzodiazépines
- VS - Mehrparteientherapievertrag für die Substitutionsbehandlung opiatabhängiger Personen

Legal texts

Federal

- SR 812.121 - Bundesgesetz vom 3. Oktober 1951 über die Betäubungsmittel und die psychotropen Stoffe (Betäubungsmittelgesetz, BetmG)
- SR 812.121.1 - Verordnung vom 25. Mai 2011 über die Betäubungsmittelkontrolle (Betäubungsmittelkontrollverordnung, BetmKV)
- SR 812.121.6 - Verordnung vom 25. Mai 2011 über Betäubungsmittelsucht und andere suchtbedingte Störungen (Betäubungsmittelsuchtverordnung, BetmSV)
- SR 812.121.11 - Verordnung des EDI vom 30. Mai 2011 über die Verzeichnisse der Betäubungsmittel, psychotropen Stoffe, Vorläuferstoffe und Hilfschemikalien (Betäubungsmittelverzeichnisverordnung, BetmVV-EDI)

Fribourg (FR)

- FR 821.0.1 - Gesundheitsgesetz (GesG) vom 16.11.1999 (Fassung in Kraft getreten am 01.07.2020)
- FR 821.20.21 - Verordnung über die Heilmittel (HMV) vom 09.03.2010 (Fassung in Kraft getreten am 01.05.2014)
- FR 821.22.11 - Verordnung über die Betäubungsmittel vom 12.04.2016 (Fassung in Kraft getreten am 01.04.2016)
- FR 821.44.4 - Gesetz über den Fonds für die Bekämpfung der Drogenabhängigkeitvom 13.02.1996 (Fassung in Kraft getreten am 01.07.2015)
- FR 821.44.12 - Beschluss über die Vorbeugung des Alkohol- und Betäubungsmittelmissbrauchsvom 07.06.1988 (Fassung in Kraft getreten am 01.01.2003)
- FR 821.44.22 - Verordnung über die kantonale Kommission für Suchtfragen vom 23.06.2014 (Fassung in Kraft getreten am 01.07.2014)
- FR 821.44.32- Beschluss über den Fonds für Forschungsarbeiten über psychische Erkrankungen und Drogenabhängigkeitvom 27.02.1996 (Fassung in Kraft getreten am 01.01.2003)

- FR 821.44.41 - Verordnung über die Verwendung des Fonds für die Bekämpfung der Drogenabhängigkeit vom 22.06.2015 (Fassung in Kraft getreten am 01.07.2015)

Geneva (GE)

- GE E 4 70 - Loi sur la création d’un fonds destiné à la luttecontre la drogue et à la prévention de latoxicomanie (LFLD) du 26 mai 1994 (Entrée en vigueur : 1er janvier 1995)
- GE K 2 05.06 - Règlement sur les institutions de santé (RISanté) du 22 août 2006 (Entrée en vigueur : 1er septembre 2006)
- GE K 4 05.12 - Règlement sur le produits thérapeutiques (RPTh) du 22 août 2006 (Entrée en vigueur : 1er septembre 2006)
- GE K 4 20.02 - Règlement relatif à l'application de la loi fédérale sur les stupéfiants et les substances psychotropes (RaLStup) du 27 juin 2007 (Entrée en vigueur : 5 juillet 2007)
- GE K 103 - Loi sur la santé (LS) du 7 avril 2006 (Entrée en vigueur : 1er septembre 2006)

Jura (JU)

- JU 810.01 - Loi sanitaire du 14 décembre 1990
- JU 810.019.2 - Arrêté portant approbation de la convention passée avec la Ligue jurassienne contre les toxicomanies du 26 avril 1990
- JU 812.21 - Loi sur la vente des médicaments du 14 décembre 1990
- JU 812.41 - Ordonnance sur les pharmacies, les produits thérapeutiques et les stupéfiants du 5 décembre 2006

Neuchâtel (NE)

- NE 800.1 - Loi de santé (LS) du 6 février 1995 (Etat au 1er janvier 2020)
- NE 804.10 - Règlement sur les produits thérapeutiques, les pharmacies et les drogeuries du 18 octobre 2006 (Etat au 1er septembre 2014)
- NE 804.30 - Règlement d'application de la loi fédérale sur les stupéfiants du 26 septembre 2001 (Etat au 1er août 2013)
- NE 821.44.32 - Arrêté instituant un Fonds de recherche en matière d'affections psychiques et de toxicodépendances du 27.02.1996 (version entrée en vigueur le 01.01.2003)

Vaud (VD)

- VD 800.01 - LOI sur la santé publique (LSP) du 29 mai 1985 (Entrée en vigueur dès le 01.09.2019)
- VD 812.11.1 - RÈGLEMENT sur les stupéfiants (RStup) du 25 mars 1987 (Entrée en vigueur dès le 20.10.1999)
- VD 818.21.4 - RÈGLEMENT sur le Groupe d'experts en matière d'addictions (RGEA) du 10 juin 2009 (Entrée en vigueur dès le 01.07.2009)
- VD 818.21.5 - RÈGLEMENT sur la Commission de promotion de la santé et de lutte contre les addictions (RCAddic) du 10 juin 2009 (Entrée en vigueur dès le 01.07.2009)

Valais (VS)

- VS 800.1 - Gesundheitsgesetz (GG) vom 12.03.2020 (Stand 01.01.2021)
- VS 812.10 - Verordnung über suchtbedingte Abhängigkeiten vom 30.05.2012 (Stand 01.10.2015)
- VS 812.200 - Heilmittelverordnung vom 04.03.2009 (Stand 01.12.2019)
